# The world's first clinical randomized trial of atrial natriuretic peptide for preventing cancer recurrence following lung cancer surgery

**DOI:** 10.1186/2050-6511-16-S1-A72

**Published:** 2015-09-02

**Authors:** Takashi Nojiri, Hiroshi Hosoda, Takeshi Tokudome, Toru Kimura, Meinoshin Okumura, Kenji Kangawa

**Affiliations:** 1 Department of Biochemistry, National Cerebral and Cardiovascular Center Research Institute, Suita, Osaka, Japan; 2 Department of General Thoracic Surgery, Osaka University Graduate School of Medicine, Suita, Osaka, Japan; 3 Department of Regenerative Medicine and Tissue Engineering, National Cerebral and Cardiovascular Center Research Institute, Suita, Osaka, Japan

## Abstract

Most patients suffering from cancer die of metastatic disease. Surgical removal of solid tumors is performed as an initial attempt to cure patients if the primary tumor meets surgical indications. However, it is possible that surgical trauma itself influences the development of early recurrence. First, resection handling of the tumor can provoke detachment of tumor cells. Second, surgical trauma provokes the severe inflammatory reaction. Vascular inflammation is considered to render the endothelium adhesive to circulating tumor cells thereby allowing the metastasis of tumor cells. We have previously reported that administration of atrial natriuretic peptide (ANP) during the perioperative period reduces inflammatory response and has a prophylactic effect on postoperative cardiopulmonary complications in lung cancer surgery. Here we demonstrate that cancer recurrence after curative surgery was significantly lower in ANP-treated patients than in control patients (surgery alone). ANP is known to bind specifically to guanylyl cyclase-A (GC-A) receptor. In mouse models, we found that metastasis of GC-A–non-expressing tumor cells (i.e., B16 mouse melanoma cells) to the lung was increased in vascular endothelium-specific*GC-A* knockout mice and decreased in vascular endothelium-specific *GC-A* transgenic mice compared with control mice. ANP inhibited the adhesion of cancer cells to human pulmonary artery endothelial cells by suppressing the E-selectin expression that is promoted by inflammation. These results suggest that ANP prevents cancer metastasis by inhibiting the adhesion of tumor cells to inflamed endothelial cells. Thus, we have planned to start a multicenter randomized clinical trial to examine the use of perioperative administration of ANP for the prevention of cancer recurrence after lung cancer surgery in this year. In the near future, we want to expand the adaptation of ANP for various types of cancer surgery.

